# Multi-site, multi-platform comparison of MRI *T*_1_ measurement using the system phantom

**DOI:** 10.1371/journal.pone.0252966

**Published:** 2021-06-30

**Authors:** Kathryn E. Keenan, Zydrunas Gimbutas, Andrew Dienstfrey, Karl F. Stupic, Michael A. Boss, Stephen E. Russek, Thomas L. Chenevert, P. V. Prasad, Junyu Guo, Wilburn E. Reddick, Kim M. Cecil, Amita Shukla-Dave, David Aramburu Nunez, Amaresh Shridhar Konar, Michael Z. Liu, Sachin R. Jambawalikar, Lawrence H. Schwartz, Jie Zheng, Peng Hu, Edward F. Jackson

**Affiliations:** 1 National Institute of Standards and Technology, Boulder, Colorado, United State of America; 2 American College of Radiology, Center for Research and Innovation, Philadelphia, Pennsylvania, United State of America; 3 University of Michigan, Ann Arbor, Michigan, United State of America; 4 NorthShore University Health System, Evanston, Illinois, United State of America; 5 St. Jude Children’s Research Hospital, Memphis, Tennessee, United State of America; 6 Cincinnati Children’s Hospital Medical Center, University of Cincinnati College of Medicine Cincinnati, Ohio, United State of America; 7 Memorial Sloan Kettering Cancer Center, New York, New York, United State of America; 8 Columbia University Medical Center, New York, New York, United State of America; 9 Washington University in St. Louis, St. Louis, Missouri, United State of America; 10 University of California, Los Angeles, California, United State of America; 11 University of Wisconsin, Madison, Wisconsin, United State of America; Linköping University, SWEDEN

## Abstract

Recent innovations in quantitative magnetic resonance imaging (MRI) measurement methods have led to improvements in accuracy, repeatability, and acquisition speed, and have prompted renewed interest to reevaluate the medical value of quantitative *T*_1_. The purpose of this study was to determine the bias and reproducibility of *T*_1_ measurements in a variety of MRI systems with an eye toward assessing the feasibility of applying diagnostic threshold *T*_1_ measurement across multiple clinical sites. We used the International Society of Magnetic Resonance in Medicine/National Institute of Standards and Technology (ISMRM/NIST) system phantom to assess variations of *T*_1_ measurements, using a slow, reference standard inversion recovery sequence and a rapid, commonly-available variable flip angle sequence, across MRI systems at 1.5 tesla (T) (two vendors, with number of MRI systems n = 9) and 3 T (three vendors, n = 18). We compared the *T*_1_ measurements from inversion recovery and variable flip angle scans to ISMRM/NIST phantom reference values using Analysis of Variance (ANOVA) to test for statistical differences between *T*_1_ measurements grouped according to MRI scanner manufacturers and/or static field strengths. The inversion recovery method had minor over- and under-estimations compared to the NMR-measured *T*_1_ values at both 1.5 T and 3 T. Variable flip angle measurements had substantially greater deviations from the NMR-measured *T*_1_ values than the inversion recovery measurements. At 3 T, the measured variable flip angle *T*_1_ for one vendor is significantly different than the other two vendors for most of the samples throughout the clinically relevant range of *T*_1_. There was no consistent pattern of discrepancy between vendors. We suggest establishing rigorous quality control procedures for validating quantitative MRI methods to promote confidence and stability in associated measurement techniques and to enable translation of diagnostic threshold from the research center to the entire clinical community.

## Introduction

Quantitative magnetic resonance imaging (qMRI) offers exciting prospects for disease detection, diagnosis, characterization, assessment of treatment response, and other applications without the need for tissue biopsy. Early work focused on *T*_1_ relaxation times to categorize different brain tumors, particularly distinguishing benign from malignant tumors. Bydder et al. observed that *T*_1_ of malignant tumors was higher than that of benign tumors [1]. Motivated by Bydder’s work, several groups tried to reproduce this observation, but had limited success due in part to technical variations [2–6]. Using the qMRI techniques available at the time, these groups found that *T*_1_ of pathologic entities/non-healthy tissue (e.g., edema, tumor) had a wide range of values, implying that *T*_1_ value would be an unreliable indicator of pathologic process or tumor grade. As a result of inconsistent findings regarding clinical value of tissue-inherent *T*_1_ values in early studies, quantitative *T*_1_ measurements were not routinely used to study tumors for many years, whereas subjective interpretation of *T*_1_-weighted imaging serves as a mainstay of clinical MRI, particularly since the introduction of exogenous contrast agents.

Recent innovations in qMRI measurement methods led to improvements in accuracy, repeatability, and acquisition speed, and have prompted renewed interest to reevaluate the medical value of quantitative *T*_1_. For example, using magnetic resonance fingerprinting, two studies found that the *T*_1_ relaxation times of glioblastoma multiforme were substantially higher compared to low grade gliomas, thus again suggesting that *T*_1_ can distinguish malignant tumors from benign tumors [7,8]. Furthermore, international consortia such as the Quantitative Imaging Biomarker Alliance (operating under the Radiological Society of North America) and the European Imaging Biomarker Alliance (sponsored by the European Institute for Biomedical Imaging Research) actively promote projects on qMRI standards and best practices for using qMRI in the clinic. These projects emphasize the use of standard objects or phantoms to assess reproducibility of measurement methods and then determine quantitative thresholds, similar to using *T*_1_ relaxation time to distinguish the grade of glioma.

Nevertheless, there remain challenges to isolate and mitigate technical sources of variability from immutable biological sources that combine to create overall variability in *T*_1_ measurements. Bojorquez et al. catalogued the broad ranges of *T*_1_ relaxation times reported in the literature for normal tissues at 3 T and observed dependence of reported *T*_1_ on the measurement method and/or MRI system [9]. Similarly, in vivo measurement studies using multiple MRI systems have varied results. Lee et al. measured *T*_1_ relaxation time in vivo across two vendor systems using a variable flip angle technique and observed high test-retest repeatability within a vendor system, but significant differences in *T*_1_ between vendors [10]. When the measurement methodology is more highly controlled, the inter-site coefficient of variation is less than 10% [11,12]. While these results are encouraging, customized pulse sequences were used across different scanner software versions in these studies [11,12], which is not representative of the typical clinical setting. This level of control is difficult to implement in multisite clinical trials and is currently not feasible for clinical settings where diverse hardware, software, and imaging protocols are to be expected.

To distinguish biological variability from technical sources that include MRI system hardware, pulse sequence design, acquisition parameters, and data reduction algorithm, a physical phantom, rather than in vivo measurements, should be used as stable reference standards for “true values” [13]. Several groups have studied *T*_1_ across measurement methods and hardware (e.g., scanner, coils) using phantoms with known *T*_1_ values in a range suitable for *T*_1_ measurement of cardiac tissue [14,15], white matter [16], or multiple tissues [17–19]. Some multi-site studies had an uneven distribution of vendor systems, which can adversely impact generalization of results. For example, Bane et al. and vanHoudt et al. both observed a site-specific dependence on the *T*_1_ measurement that may be dependent on the distribution of systems included in their studies [17,19].

The purpose of this study was to determine the variability in *T*_1_ measurements on a variety of MRI systems to ascertain the feasibility of applying diagnostic threshold *T*_1_ measurements across multiple clinical sites. We used the International Society of Magnetic Resonance in Medicine/National Institute of Standards and Technology (ISMRM/NIST) system phantom [20] to assess variations of *T*_1_ measurements across MRI systems at 1.5 tesla (T) (two vendors, with number of MRI systems n = 9) and 3 T (three vendors, n = 18).

## Methods

### Image acquisition

Two ISMRM/NIST system phantoms from the same production run were imaged at multiple sites on systems from three vendors (General Electric (GE) Healthcare Systems, Waukesha, WI, USA; Siemens Healthcare, Erlangen, Germany; and Philips, Best, The Netherlands) at 1.5 tesla and 3 tesla using head coils with 8 to 32 channels (Table 1). At 1.5 T, there were four GE Medical Systems and five Siemens systems, and at 3 T there were six GE Medical Systems, five Philips, and seven Siemens systems.

**Table 1:** MRI system details.

Index	Field (T)	Frequency (MHz)	Manufacturer	Scan Dates	System	Software	Head Coil	Phantom
1	3	127.77	Philips	2015-05-19	Ingenia	5.1.7	32-ch receive	B
2	3	127.77	Philips	2015-07-20	Ingenia	5.1.7	32-ch receive	B
3	3	127.77	Philips	2015-07-31	Ingenia	5.1.7	32-ch receive	B
4	3	123.18	Siemens	2015-05-12	Verio	syngo MR D13	12-ch receive	A
5	3	123.18	Siemens	2015-07-20	Verio	syngo MR D13	12-ch receive	A
6	3	123.18	Siemens	2015-07-27	Verio	syngo MR D13	12-ch receive	A
7	3	127.76	GE Medical systems	2015-10-22	SIGNA Discovery MR750w	DV25	8-ch receive	A
8	3	127.76	GE Medical systems	2015-10-23	SIGNA Discovery MR750w	DV25	8-ch receive	A
9	3	127.76	GE Medical systems	2015-10-29	SIGNA Discovery MR750w	DV25	8-ch receive	A
10	3	123.24	Siemens	2015-08-12	Prisma	syngo MR D13D	20-ch receive	A
11	3	123.24	Siemens	2015-08-13	Skyra	syngo MR E11	20-ch receive	A
12	3	127.75	Philips	2015-08-17	Ingenia	5.1.9	32-ch receive	B
13	3	127.74	GE Medical systems	2015-08-29	SIGNA Discovery MR750w	DV25	24-ch receive	B
14	3	123.26	Siemens	2015-10-02	Prisma	syngo MR D13D	20-ch receive	A
15	3	123.25	Siemens	2015-10-03	Prisma	syngo MR D13D	20-ch receive	B
16	3	127.61	GE Medical systems	2015-12-01	SIGNA Discovery MR750w	DV24	24-ch receive	A
17	3	127.76	Philips	2015-12-11	Ingenia CX	5.1.8	32-ch receive	B
18	3	127.72	GE Medical systems	2016-03-06	SIGNA HDxt	HD23	8-ch receive	A
19	1.5	63.62	Siemens	2015-05-20	Avanto	syngo MR D13	12-ch receive	A
20	1.5	63.62	Siemens	2015-08-14	Avanto	syngo MR B17	12-ch receive	A
21	1.5	63.64	Siemens	2015-08-14	Avanto	syngo MR B17	12-ch receive	A
22	1.5	63.85	GE Medical systems	2015-06-05	SIGNA HDxt	HD23	8-ch receive	A
23	1.5	63.85	GE Medical systems	2015-08-29	SIGNA HDxt	HD23	8-ch receive	B
24	1.5	63.64	Siemens	2015-09-30	Avanto	syngo MR B17	12-ch receive	A
25	1.5	63.66	Siemens	2015-10-03	Avanto	syngo MR D13B	20-ch receive	B
26	1.5	63.86	GE Medical systems	2016-02-27	SIGNA Optima MR450w	DV25	24-ch receive	A
27	1.5	63.88	GE Medical systems	2016-03-07	SIGNA HDxt	HD16	8-ch receive	A

The two phantoms included in this study were prepared in collaboration between NIST and CaliberMRI (Boulder, CO, USA) using solutions prepared by NIST. The two phantoms were precision machined using identical protocols and contained the same solutions. The large number of samples with prescribed concentration variations allows for identification and elimination of defective samples. The phantoms were shipped via overnight service between sites after imaging was complete. Table 1 indicates which phantom was imaged at each location. The focus of this study was the NiCl_2_ array (previously called the *T*_1_ array) in the ISMRM/NIST system phantom. The NiCl_2_ array was chosen since it has a smaller temperature and field dependence than other available reference arrays [20]. The NiCl_2_ array contains 14 spheres that are doped with varying concentrations of NiCl_2_ to achieve a $\sqrt{2}$ progression of *T*_1_ values from approximately 20 ms to 2000 ms at 1.5 T. The reference *T*_1_ times at 1.5 T and 3 T were determined using the NMR-based relaxation time measurement service provided by NIST. These measurements are traceable to the international system of units and values and have a 3*σ* uncertainty of less than 1.5% (the real value has a > 99.7% probability of being within ± 1.5% of the reference value). Measurement details are available [21].

MRI-based *T*_1_ relaxation time was measured using two methods: inversion recovery (IR) using 2D fast spin echo inversion recovery, and variable flip angle (VFA) using 3D fast spoiled gradient echo. Detailed parameters defining the scan protocols are provided in Table 2 for IR and Table 3 for VFA. In addition to the details in Tables 2 and 3, sites were given detailed instructions, including photos of the phantom in a head coil and example images to convey the phantom placement and imaging protocols. For VFA data, participants were instructed to set signal gains by performing a prescan using a 15-degree flip angle; system settings were fixed for subsequent scans to the extent possible. Potential variable signal scaling across series was accounted for in image analysis [22].

**Table 2:** Inversion recovery (IR) measurement protocols.

*T*_1_—VTI Series	GE Medical Systems	Philips	Siemens
**Sequence**	2D/FSE-IR	2D/IR-SK	2D/TSE-IR
**Scan Plane**	Coronal	Coronal	Coronal
**Scan Options**	EDR (Extended Dynamic Range)	2D IR; Fast = TSE (Turbo Spin Echo)	
**Section Thickness/Gap (mm)**	6	6	6
**TR (ms)**	4500	4500	4500
**TE (ms)**	Min Full (7.6)	7	6.9
**TI Values (ms)**	50, 75, 100, 125, 150, 250, 1000, 2000, 3000	35, 75, 100, 125, 150, 250, 1000, 1500, 2000, 3000	35, 75, 100, 125, 150, 250, 1000, 1500, 2000, 3000
**Echo Train Length (ETL)**	3	6	6
**Number of Averages**	1	1	1
**Matrix (Frequency Encode)**	256	256	256
**Matrix (Phase Encode)**	192	252	192
**Matrix (Slice Encode)/# of Slices**	1	1	1
**Pixel Bandwidth (Hz)**	391	436	279
**Bandwidth (kHz)–GE only**	50		
**FOV (FE, mm)**	250	250	250
**FOV (PE, mm)**	200 (0.8 PFOV)	250	250
**Pixel Size (mm x mm)**	0.98 x 0.98	0.98 x 0.98	0.98 x 0.98
**Phase Encode Direction**	RL	RL	RL
**Notes**	Minimum TI allowed on GE: 50ms Autoprescan with TI = 50 ms	Reconstruct Magnitude, Real, Imaginary images. Do “FullPrep” for each of these ten series.	Use SOS (sum-of-squares) multi-channel coil reconstruction, not default ACC (adaptive coil combine).
**Series**	T1 VTI	T1 VTI	T1 VTI
**Approximate Acquisition Time per TI Setting (min)**	4.0	3.5	3.02
**# of TI Settings**	9	10	10
**Total Time for this Series (min)**	36.0	35.0	30.20

**Table 3:** Variable flip angle (VFA) measurement protocols.

*T*_1_—VFA Series	GE Medical Systems	Philips	Siemens
**Sequence**	3D/FSPGR	3D/SPGR	3D/RF spoiled GRE
**Scan Plane**	COR	COR	COR
**Scan Options**	EDR/Z2	3D FFE; Fast = none	
**Section Thickness/Gap (mm)**	3/0	6/0	6/0 (or 3/0 if error)
**TR (ms)**	Min (6.0)	6.6	6.6
**TE (ms)**	Min (1.4)	1.8	2.44
**TI Values (ms)**			
**Flip Angle (deg)**	2, 5, 10, 20, 25, 30	2, 5, 10, 20, 25, 30	2, 5, 10, 20, 25, 30
**ETL (Echo Train Length)**	1	1	1
**Number of Averages**	4	4	4
**Matrix (Frequency Encode)**	256	256	256
**Matrix (Phase Encode)**	192	192	192
**Matrix (Slice Encode)/# of Slices**	34	28	32
**Pixel Bandwidth (Hz)**	488	904	280
**Bandwidth (kHz)–GE only**	62.5		
**FOV (FE, mm)**	250	250	250
**FOV (PE, mm)**	250	250	250
**Pixel Size (mm x mm)**	0.98 x 0.98	0.98 x 0.98	0.98 x 0.98
**Phase Encode Direction**	RL	RL	RL
**User CVs**	Turbo = 0		
**Notes**	1) Yields 30 3-mm sections. 2) Autoprescan with FA = 15. 3) Ensure gain settings do not vary between series, i.e., use Manual Prescan. 4) Include fiducial spheres above & below T1 spheres in scan volume. 5) Using Turbo = 0 should maintain the same TE/TR for each FA.	1) Reconstructed at 3 mm. 2) Ensure gain settings do not vary between series, i.e., use MPS. Autoprescan using 15 deg FA. 3) Include fiducial spheres above & below T1 spheres in scan volume.	1) Reconstructed at 3 mm. 2) Ensure gain settings do not vary between series, i.e., use MPS. Autoprescan using 15 deg FA. 3) Include fiducial spheres above & below T1 spheres in scan volume.
**Series**	T1 VFA	T1 VFA	T1 VFA
**Approximate Acquisition Time per Flip Angle Setting (min)**	2.6	3.0	1.23
**# of Flip Angle Settings**	7	7	7
**Total Time for this Series (min)**	18.2	21.0	8.61

The protocol did not require that the phantom be placed in the scan room for temperature equilibration prior to measurement. The phantom temperature was measured before and after imaging using a NIST-traceable, calibrated thermometer (Control Company, Friendswood, TX, USA) placed within the phantom by removing the top screw of the phantom. Incorrect temperature measurement (e.g., measuring the temperature of the room rather than the temperature of the phantom) did not require reacquisition of the data. Temperature changes are not expected to impact our study, as *T*_1_ times for NiCl_2_ are known to be relatively insensitive to temperature over the range 16°C to 26°C, and the 10 highest NiCl_2_ concentration spheres have less than ± 4% variation over this range [20].

### Image analysis and selection of regions-of-interest

Two observers performed centralized quality control on all submitted data to ensure adherence to the prescribed imaging protocol with both observers reviewing all data. Deviation from the acquisition protocol resulted in submission rejection (e.g., incorrectly setting the signal gains for the VFA experiment). Sites were encouraged to repeat the image acquisition correctly; four image sets were initially rejected and then properly acquired.

**Fig 1:**
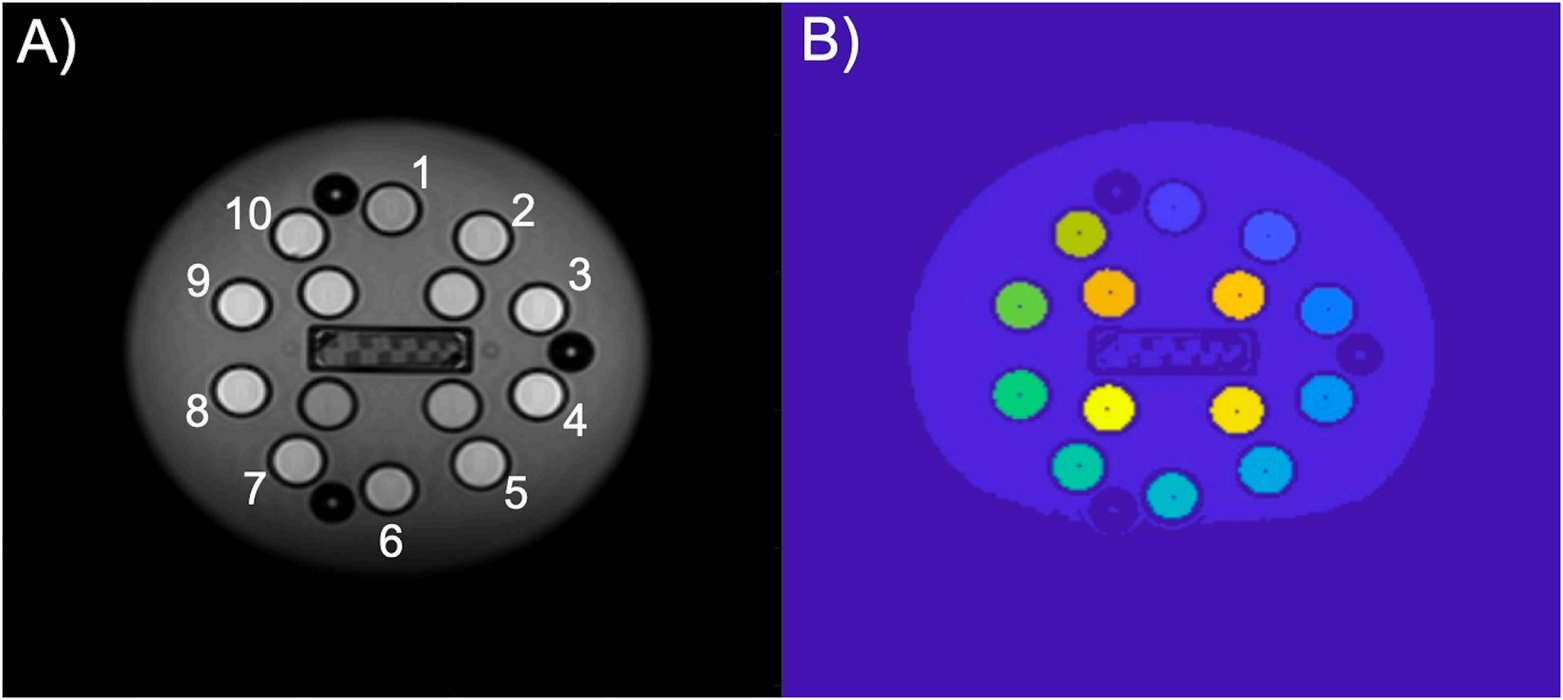
An example coronal slice of the ISMRM/NIST system phantom through the NiCl_2_ array and resulting segmentation. (A) The shortest inversion time image used for identification of sample spheres and (B) the segmentation with sample sphere centers identified.

We used special-purpose, automated segmentation software to identify the 14 spheres containing *T*_1_ samples (“sample spheres”) and then select the regions-of-interest (ROIs) for analysis (Fig 1). We performed this segmentation in the shortest inversion time (TI) image in the IR image stack, as this image generally provided the most contrast between sample spheres and the phantom background (water). Likewise, the protocol required that the VFA scans take place immediately after the IR scans with no repositioning of the phantom. Thus, the ROIs determined for the IR measurement were the same in the VFA data analysis from the same scan session.

The NiCl_2_ (i.e., *T*_1_) array consists of 14 spheres, each with an inner radius of 7.5 mm. For each of these spheres, the regions of interest were defined as the collection of pixels within each sphere and well-separated from the boundary. Previous publications describe the details of the ROI identification algorithm [18] and [20]. In brief, we applied a gradient filter to the measured image, then thresholded the result to define a binary image of region edges. Next, we used an optimization routine to determine the rigid transformation—translation and rotation—such that the sample spheres of the known phantom array covered the edge pixels determined in the first step. The results of this rigid transformation served to initialize an iterative process to refine the center of each sample sphere individually. This step accommodated geometric distortions introduced by the scanner. With the centers of all 14 sample spheres thus determined in the measurement frame, we defined the ROI as all pixels falling within 4 mm of this center point (well within the interior of each sample sphere). At the resolution of these images, the result is that each ROI consisted of approximately 52 pixels. The mean intensity value of these pixels defined the signal value corresponding to that ROI for the given TI or flip angle (IR and VFA, respectively). The ROI identification software is part of the qMRLab suite [23,24] and can be provided by the authors upon request. The data in this study will be available at doi:10.18434/mds2-2357.

Prior to *T*_1_ data analysis, we rescaled images from Philips systems as specified by Chenevert et al. [22]. The segmentation code and *T*_1_ data analysis code were written and performed using MATLAB (The MathWorks, Inc., Natick, MA, USA).

### T_1_ data analysis

Inversion recovery and variable flip angle are two qMRI protocols for *T*_1_ measurement. In both protocols, *T*_1_ arises as a parameter in a model for the measured MR signal intensities as a function of an experimental variable—inversion time (*TI*_*k*_) in IR experiments and flip angle (*α*_*m*_) for VFA [25].

The measurement model for the IR experiment is

$$y_{k}=|M_{0}\frac{1-\text{cos}(\theta_{180})e^{-\frac{TR}{T_{1}}}-(1-\text{cos}\left(\theta_{180}\right))e^{-\frac{{TI}_{k}}{T_{1}}}}{1-\text{cos}(\theta_{180})\text{cos}\left(\theta_{90}\right)e^{-\frac{TR}{T_{1}}}}|+n_{k}.$$ (1)

Here *y*_*k*_ is the measured signal at the *k*-th inversion time, *M*_0_ is the initial magnitude of the magnetization signal, and *n*_*k*_ represents measurement noise. In addition to *TI*_*k*_, the fixed experimental parameters are: *TR*, the relaxation time, and *θ*_180_, *θ*_90_, the flip angles. In principle, for a given set of *TI*_*k*_ and associated values *y*_*k*_, one could attempt to invert the above equation for all parameters. However, as our objective is to estimate *T*_1_ alone, and we combine terms and fit the IR signal to a general exponential model:

$$y_{k}=|A{+Be}^{-\frac{{TI}_{k}}{T_{1}}}|+n_{k}.$$ (2)

Here, the constants *A* and *B* are required for mathematical consistency but may not have a physical interpretation in all cases. Fitting data using non-linear least squares is a natural approach as it corresponds to the maximum likelihood estimator in the case that the noise variables *n*_*k*_ are independent, identically distributed Gaussians. However, the absolute value appearing in the IR signal model entails a loss of differentiability at measurement points where the signal is near zero. To avoid this, we modified the objective and solved the following non-linear least squares problem to estimate *T*_1_, *A* and *B*:

$${\text{min}}_{T_{1},\;A,\;B}\sum_{k=1}^{K}{\left(y_{k}^{2}-\;{\left(A+Be^{-\frac{{TI}_{k}}{T_{1}}}\right)}^{2}\right)}^{2}.$$ (3)

We solved this smooth problem via Newton iterative refinement of an initial guess found by a search over a dense grid in the three-dimensional parameter space (*T*_1_, *A*, and *B*). Note that the residuals (Eq 3) were never zero due to measurement noise and also to signal not accounted for by the model. We ran Newton iterations until the changes in the residuals were orders of magnitude less than the residuals themselves. In principle, one could use the stationary point of the smooth problem as an initial guess for the original, non-smooth problem involving absolute values. Generally, we found the *T*_1_ values to not be substantially different. However, this could be a topic for future investigation.

The analysis of VFA data proceeded along similar lines. In this case, we modeled the measured MRI signal as a function of flip angle by the Ernst equation (see [26] or, for example, [27])

$$z_{m}=M_{0}\text{sin}\alpha_{m}\frac{1-e^{-\;\frac{TR}{T_{1}}}}{1-e^{-\;\frac{TR}{T_{1}}}\text{cos}\alpha_{m}}+n_{m},$$ (4)

where *z*_*m*_ is the measured signal at the flip angle *α*_*m*_, *TR* is a fixed experimental parameter, *n*_*m*_ is measurement noise, and *M*_0_ is the signal corresponding to the ROI equilibrium magnetization. Once again, estimates of *T*_1_ and *M*_0_ are determined by non-linear least squares minimizing the sum:

$${\text{min}}_{{T_{1},\;M}_{0}}\sum_{m=1}^{M}{\left(z_{m}-M_{0}\text{sin}\alpha_{m}\frac{1-e^{-\;\frac{TR}{T_{1}}}}{1-e^{-\;\frac{TR}{T_{1}}}\text{cos}\alpha_{m}}\right)}^{2}.$$ (5)

As above, we determined initial values of *T*_1_ and *M*_0_ by grid search and refined these by Newton iteration.

### Statistical methods

We compared the *T*_1_ measurements from IR and VFA scans to phantom reference values obtained by NIST’s MRI Biomarker Measurement Service based on gold-standard NMR [21]. This service provides measurements with less than 1.5% error traceable to the international system of units; we refer to these NMR measurements as “true values” [28] and indicate them by *T*_1,NMR_. We used Analysis of Variance (ANOVA) to test for statistical differences between *T*_1_ measurements grouped according to MRI scanner manufacturers and static field strengths. We referred to such groupings as “vendor” and “field” respectively. We performed all analyses using the Statistical Toolbox within MATLAB (The MathWorks, Inc., Natick, MA, USA).

As true values of *T*_1_ span two orders of magnitude, we performed our analysis on normalized errors to create a uniform scale for all measurements. For each ROI in the NiCl_2_ array, we define the normalized measurement error as

$$\tilde{{\Delta T}_{1}}=\frac{T_{1}-T_{1,NMR}\;}{T_{1,NMR}}\times 100\%.$$ (6)

We conducted all hypothesis tests on various pooled averages of this normalized deviation.

Our statistical analysis tested the null hypothesis that the mean normalized measurement errors were the same for all groups. The hypothesis test for normalized group mean differences was performed using the **anovan** function in MATLAB. A two-way ANOVA analysis indicated significant interactions between vendor and field grouping variables. As a result, we used a simple main effects model [29–31], considering the data from the two field values (1.5 T and 3 T) separately. We analyzed the pairwise differences between group means using the **multcompare** command with Tukey-Kramer’s honestly significant difference statistics. The confidence level for all statistical tests was *α* = 0.05.

## Results

**Fig 2:**
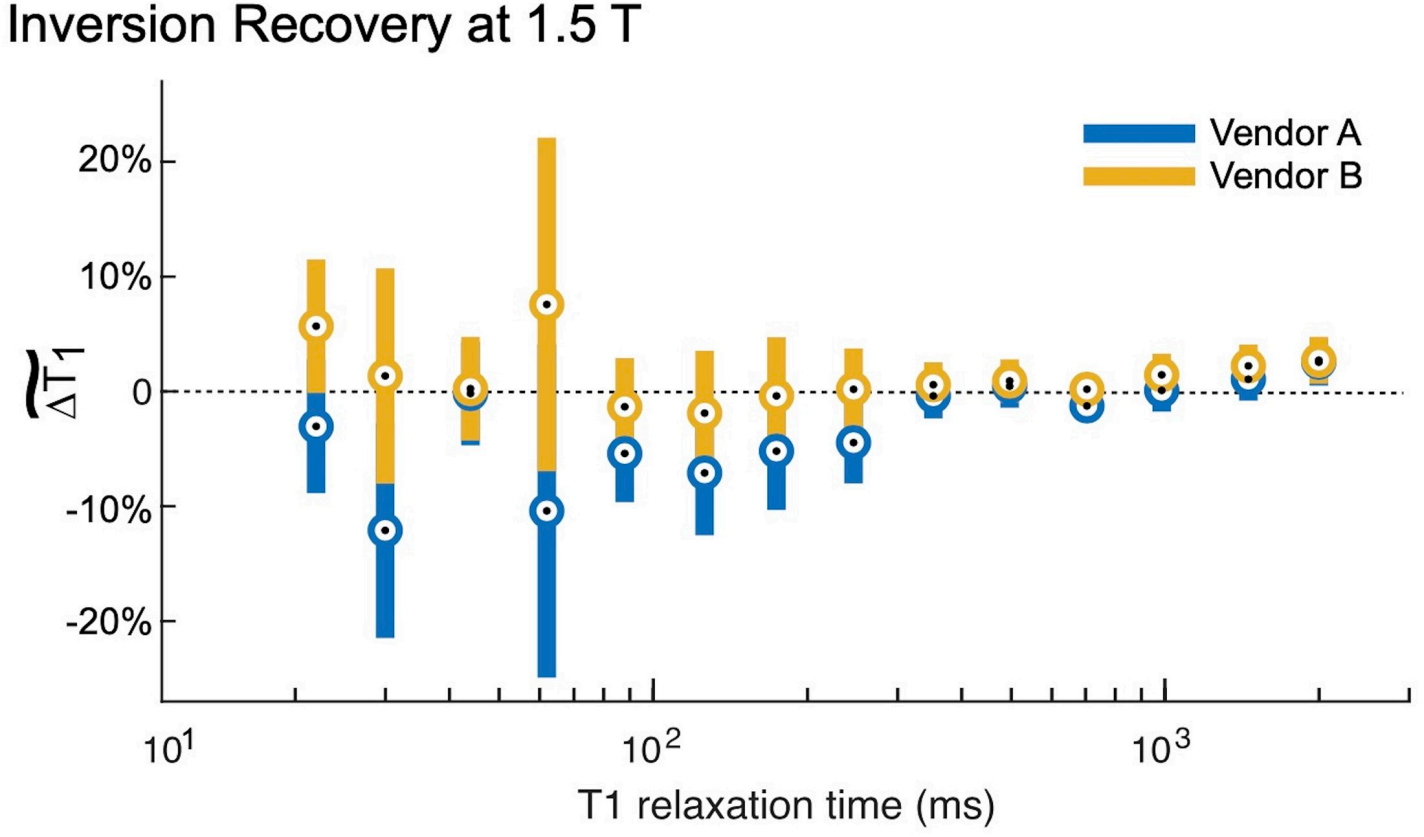
Inversion recovery measurements at 1.5 T. The inversion recovery (IR) measurements at 1.5 T both over- and underestimated the *T*_1,NMR_. The circles represent the within group means, and the error bars are 95% confidence intervals about these means. The IR measurements, especially in the range of physiological *T*_1_ values (~250 ms for adipose tissue to 1800 ms for grey matter) are biased approximately 5% high. Both vendors exhibited this bias; there are no significant differences between them throughout the entire range of *T*_1_ times spanned by the ISMRM/NIST phantom array (Table 4).

**Fig 3:**
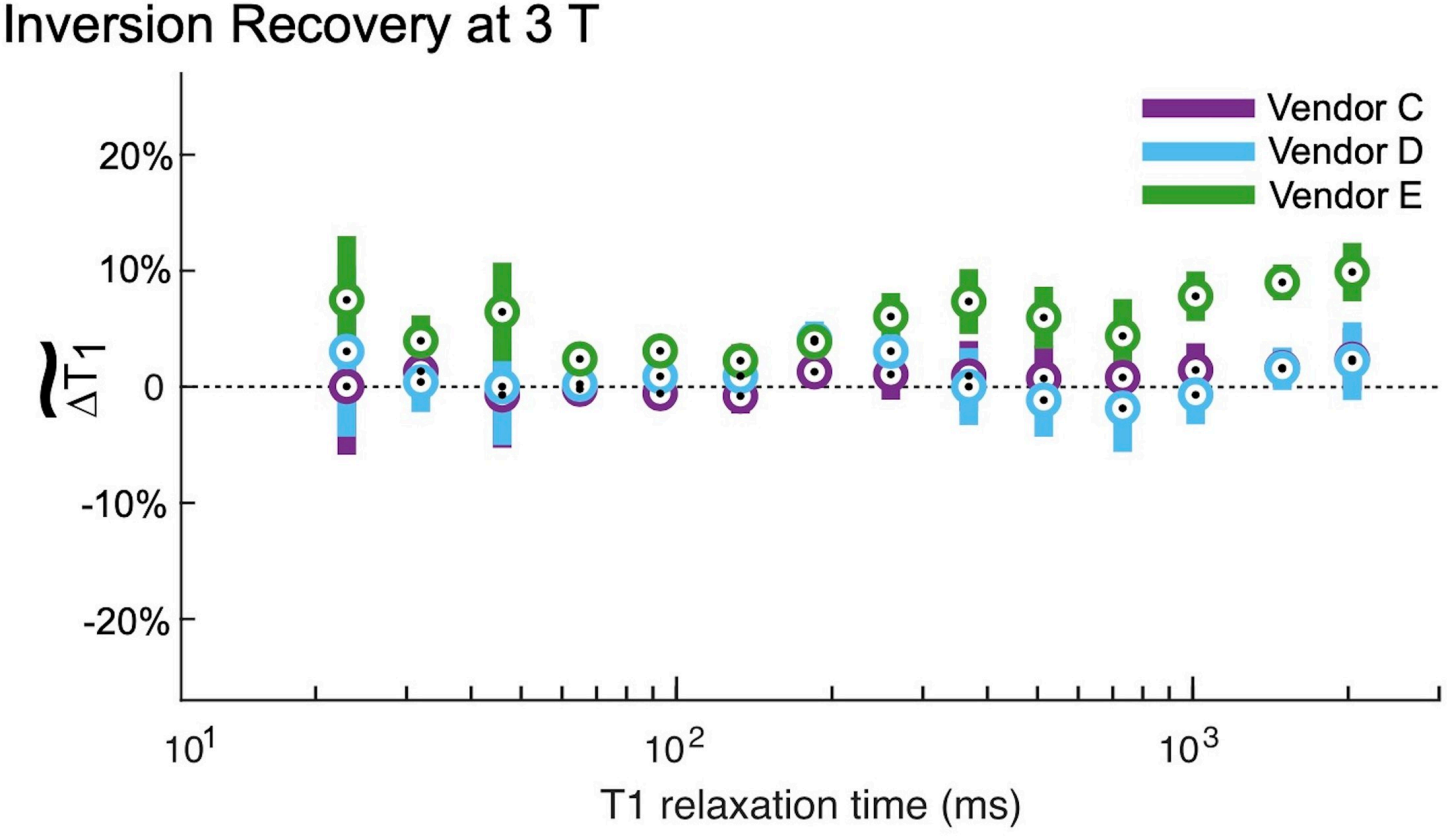
Inversion recovery measurements at 3 T. At 3 T, the inversion recovery (IR) measurements generally overestimated the *T*_1,NMR_. The circles represent the within group means, and the error bars are 95% confidence intervals about these means. There were no differences between vendors C and D. By contrast, vendor E is biased almost 10% higher than vendors C and D for *T*_1_ values in the physiologically relevant range. Please see Table 5 for tests of significance.

The IR method had minor deviations from the NMR-measured *T*_1_ value at both 1.5 T and 3 T (Figs 2 and 3). At both field strengths, the IR method both over- and underestimated the true *T*_1_ as indicated by the positive and negative bias in the figures. At 1.5 T, there were no statistically significant differences between vendors (Table 4). At 3 T, Vendor E is biased higher than Vendors C and D with significant differences (Table 5) over a true *T*_1_ range of 65 ms to 2033 ms. This range of *T*_1_ times spans multiple tissue types, including white matter, grey matter, muscle, myocardium, prostate, and fibroglandular tissues.

**Table 4:** ANOVA comparison for IR, VFA at 1.5 T.

*T*_1,NMR_ (ms)	Vendor– 1.5 T	
	A v. B	
	IR	VFA
1955	0.9250	0.1425
1454	0.4846	0.1675
985	0.4166	0.2433
704	0.2302	0.2123
496	0.7794	0.1613
352	0.5785	0.1406
246	0.1664	0.1371
174	0.3039	0.1596
126	0.2943	0.1793
88	0.2933	0.1475
62	0.1867	0.1276
44	0.9146	0.0542
30	0.1338	**0.0355**
22	0.1163	**0.0136**

p-value for ANOVA comparison with 95% confidence interval testing for IR, VFA differences between vendors at 1.5 T, considering each *T*_1,NMR_ value individually. A low value indicates rejection of the null hypothesis that mean values of the two groups are the same.

**Table 5:** ANOVA comparison for IR, VFA at 3 T.

*T*_1,NMR_ (ms)	Vendor– 3 T					
	IR			VFA		
	C v. D	C v. E	D v. E	C v. D	C v. E	D v. E
2033	0.9970	**0.0056**	**0.0109**	**0.0119**	0.9712	**0.0061**
1489	0.9980	**0.0001**	**0.0001**	**0.0166**	0.8883	**0.0055**
1012	0.4999	**0.0056**	**0.0008**	**0.0106**	0.8952	**0.0036**
731	0.6117	0.3649	0.0828	**0.0037**	0.9552	**0.0017**
514	0.7037	0.0646	**0.0163**	**0.0012**	0.9984	**0.0008**
368	0.9247	**0.0299**	**0.0184**	**0.0007**	0.9600	**0.0003**
260	0.5113	**0.0201**	0.2208	**0.0031**	0.7508	**0.0006**
185	0.0637	0.0628	0.9783	**0.0271**	0.5641	**0.0031**
133	0.3646	**0.0401**	0.5122	0.2006	0.3089	**0.0123**
93	0.1293	**0.0001**	**0.0163**	0.4240	0.1926	**0.0199**
65	0.8806	**0.0178**	0.0628	0.2636	0.3983	**0.0251**
46	0.9795	0.1217	0.2024	0.3201	0.6593	0.0741
32	0.8823	0.3176	0.1665	0.0505	0.8880	**0.0180**
23	0.8240	0.2355	0.6464	**0.0064**	0.9861	**0.0037**

p-value for ANOVA comparison with 95% confidence interval testing for IR, VFA differences between vendors at 3 T, considering each *T*_1,NMR_ value individually.

**Fig 4:**
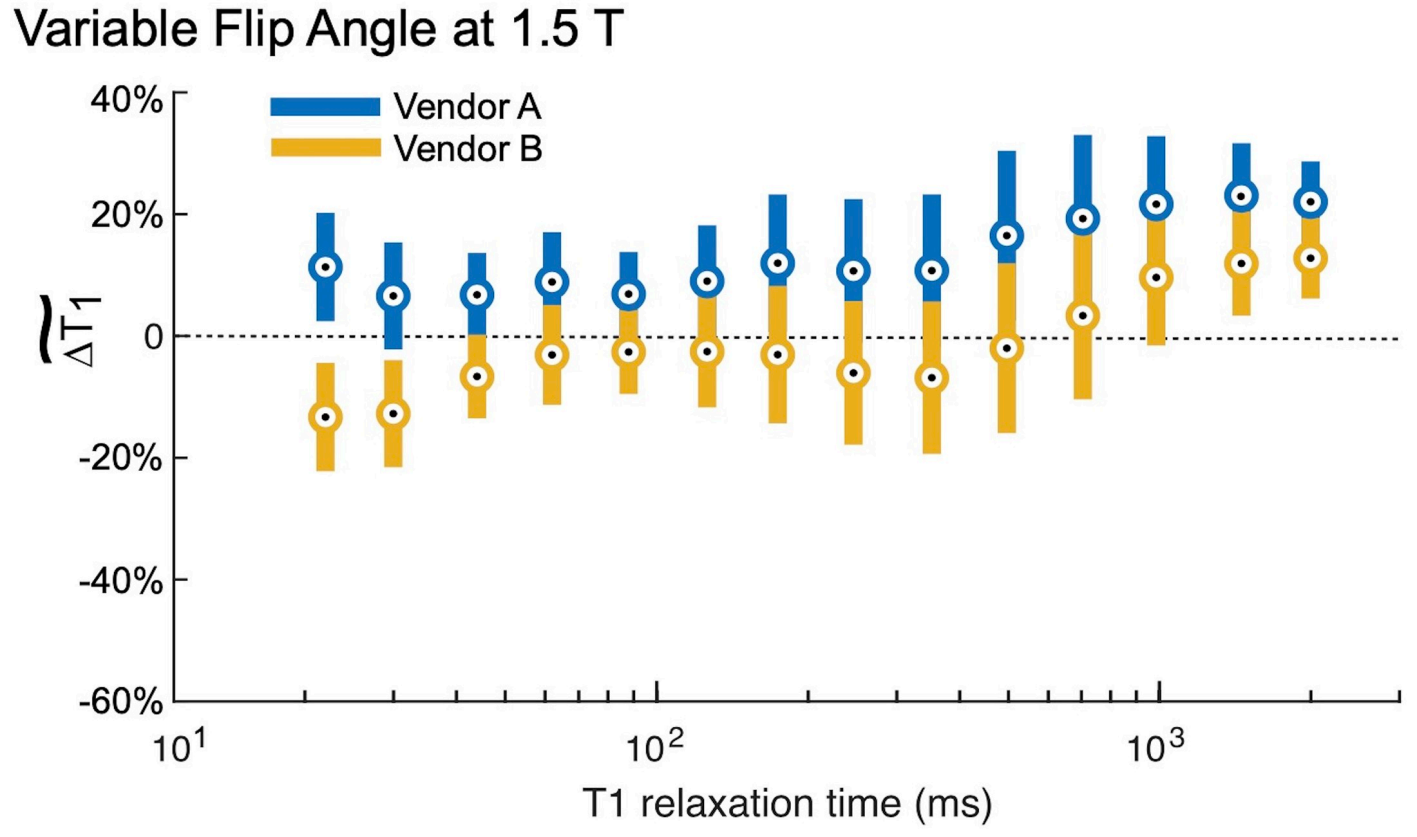
Variable flip angle measurements at 1.5 T. The variable flip angle (VFA) measurements at 1.5 T had a broader range of deviations than the IR measurements (Fig 2), and again both over- and underestimated the *T*_1,NMR_. The circles represent the within group means, and the error bars are 95% confidence intervals about these means. There were significant (95% CI) differences between Vendors A & B for the two shortest *T*_1_ relaxation times; however, the *T*_1_ relaxation time of those spheres is below those values typically measured in the body.

**Fig 5:**
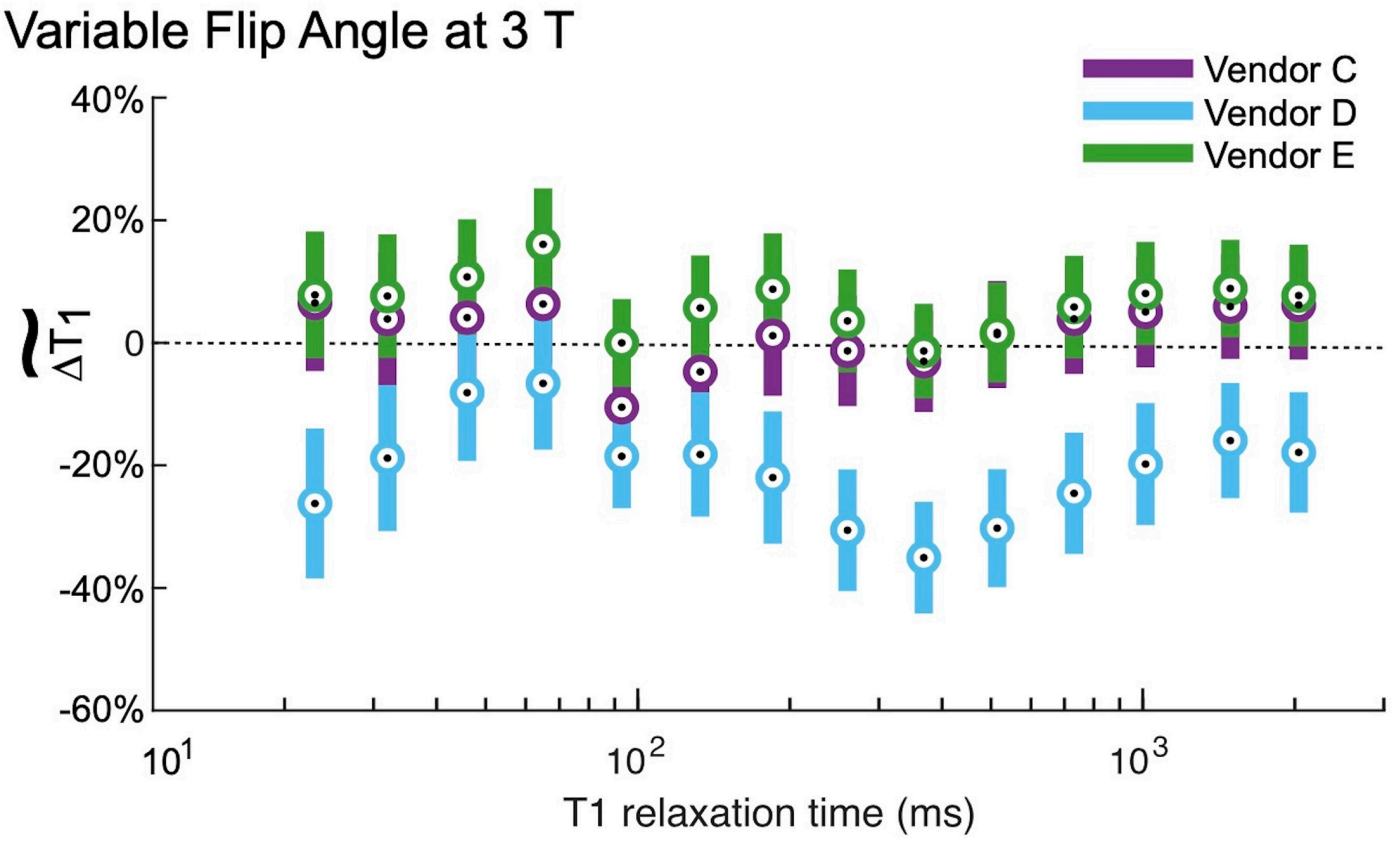
Variable flip angle measurements at 3 T. At 3 T, the variable flip angle (VFA) measurements had a much broader range of deviations than the IR measurements (Fig 3). The circles represent the within group means, and the error bars are 95% confidence intervals about these means. Vendors C and D and D and E are significantly (95% CI) different for many spheres; p-values are given in Table 5. Vendors C and E generally overestimated the *T*_1,NMR_, while vendor D underestimated it. Finally, we observe a pattern in the vendor D deviation: The greatest deviation (largest underestimation) is for samples with *T*_1_ relaxation times 260 ms, 368 ms, 514 ms, which are located in the “chin” of the phantom (Fig 1, sample spheres 5–7).

**Fig 6:**
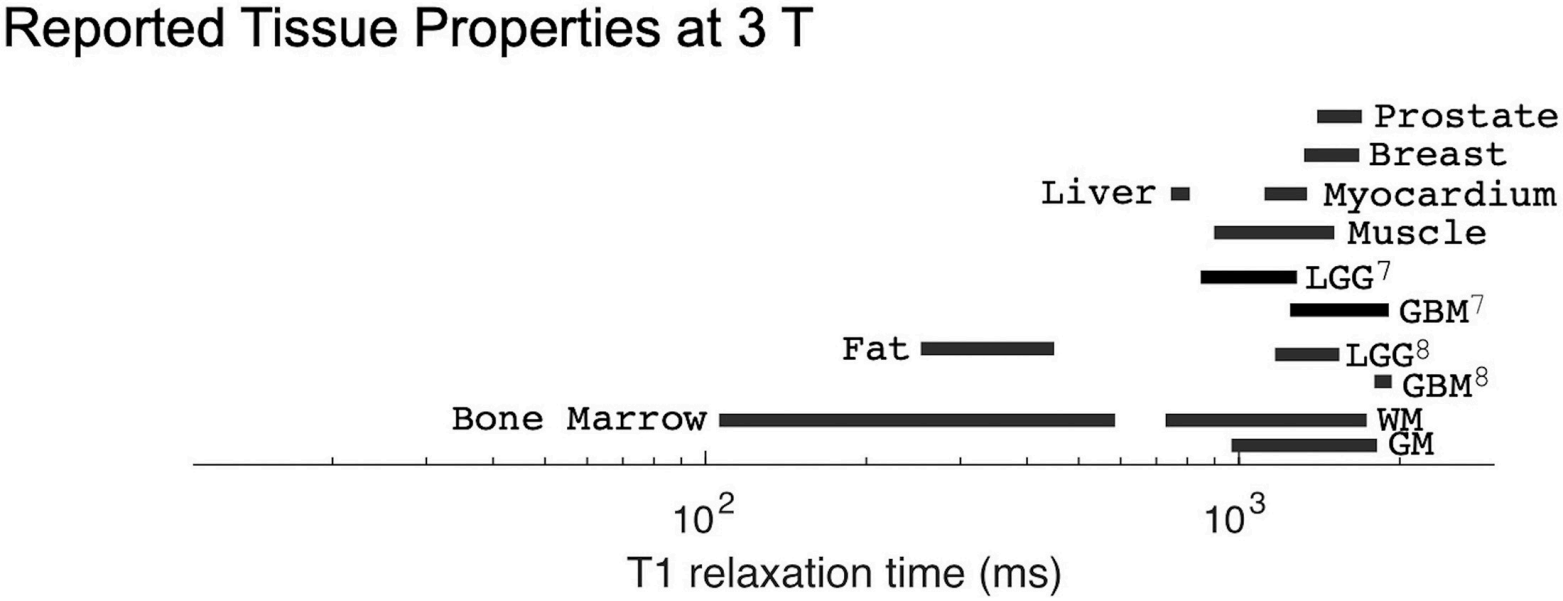
Reported tissue properties at 3 T. Physiological values of normal and diseased tissue from [7–9]. Unless otherwise noted by a superscript, the reference is [9].

The VFA measurements of *T*_1_ exhibited substantially more bias and less reproducibility than using IR. The relative errors for each field strength and vendor are shown in Figs 4 and 5. Note that the vertical axes for these plots span twice the range as for the corresponding IR figures. At 1.5 T, VFA has a broader range of deviation than IR, but the only significant differences between vendors A and B occur at very short *T*_1_ times (Table 4). By contrast, at 3 T, the VFA measurements for vendor D are significantly different than the other two vendors (C, E) for most of the samples throughout the clinically relevant range (examples of physiological values are given in Fig 6). The bias is unpredictable as vendor D underestimates the *T*_1_ value while vendors C and E overestimate it. Finally, there is a variation in the errors correlated with spatial position of the ROIs situated within the phantom. This effect manifests as an oscillation visible in VFA measurements for all field values and vendors. However, it is most pronounced at 3 T for vendor D. The four samples with the shortest *T*_1_ values are arranged in a square grid in the center of the phantom, and the remaining ten samples are placed in a circle around the outside of the phantom (Fig 1). The vendor D sample with the largest underestimation of *T*_1_ is located approximately at the “chin” (Fig 1; sample spheres 5–7).

**Fig 7:**
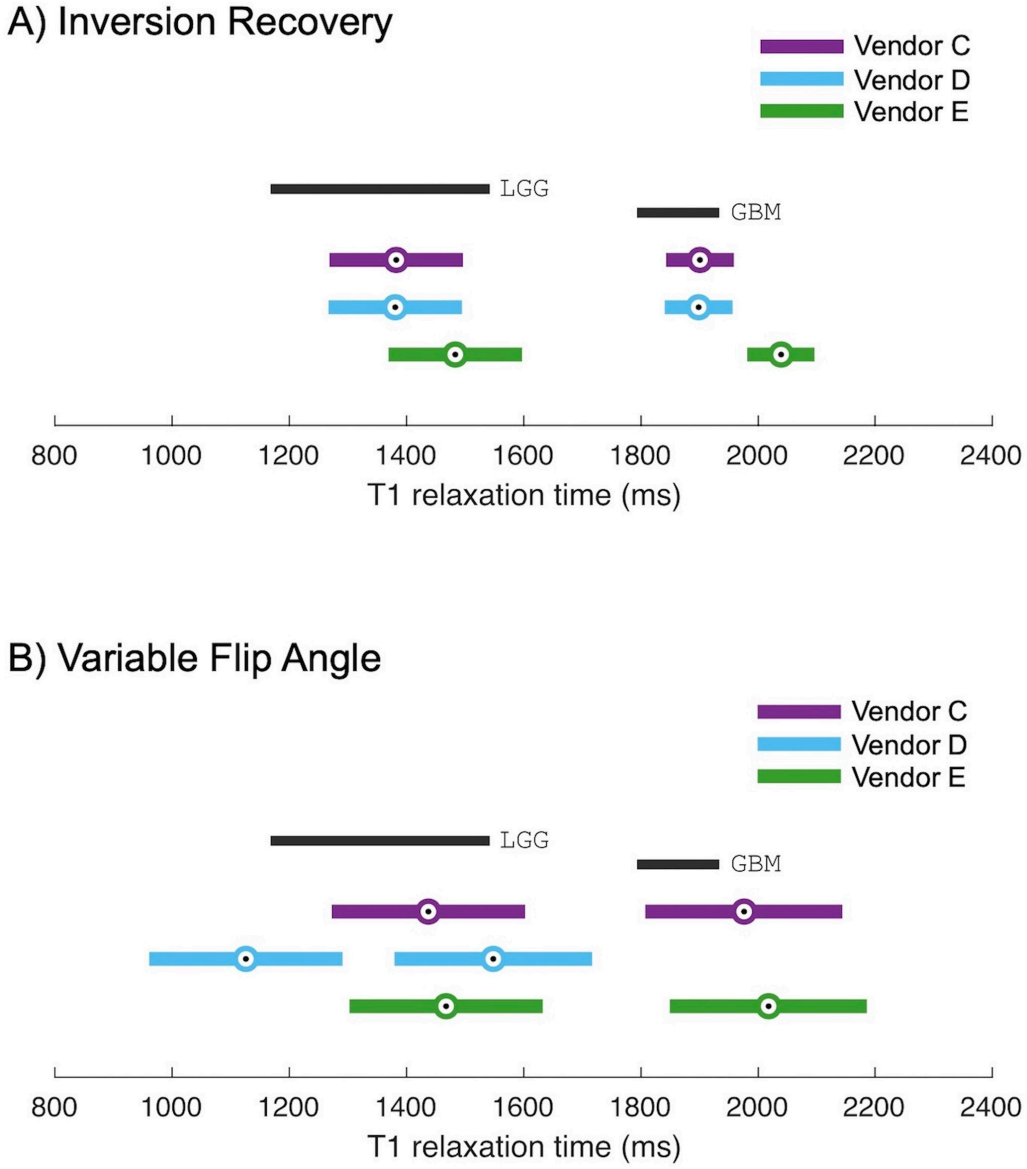
Impact of vendor differences in T1 measurement. Here, we have plotted the reported *T*_1_ of low grade glioma and glioblastomas [8] and an estimate for each vendor system of the diagnostic range for low grade glioma and glioblastomas based on the bias and dispersion of that system. The challenge is to define a diagnostic criterion based on *T*_1_ to distinguish low grade glioma from glioblastoma that would be suitable across vendor systems. If *T*_1_ relaxation time is measured using IR (A), the overestimate of values by vendor E is small compared to the range of physiological values, and as a result, *T*_1_ measured by IR could be a reliable measure across vendor systems. However, if the VFA method is used (B), the underestimate of *T*_1_ on vendor D could inaccurately diagnose a glioblastoma as a low-grade glioma, an incorrect determination with serious impacts to patient management.

Finally, we illustrate how these vendor differences could potentially impact clinical diagnostics. Consider a scenario in which *T*_1_ measurements are used to distinguish between low grade glioma (LGG) and glioblastoma multiforme (GBM). In a previous study, de Blank et al. indicated that at 3 T, LGG tissue can be characterized as having a *T*_1_ of 1355 ms ± 187 ms whereas GBM tissue has a *T*_1_ of 1863 ms ± 70 ms [8]. The range of *T*_1_ times associated with these tissues are shown in Figs 6 and 7. This range of *T*_1_ times is approximately covered by spheres 1 and 2 of the NiCl_2_ array (2033 ms and 1489 ms, respectively). For *T*_1_ times spanned by these two spheres, we assume that the relative bias and dispersion are constant for all measurement modalities and vendors. From Fig 3, for IR measurements at 3 T, we estimated these relative biases and dispersions to be: 2% positive bias for vendors D and E, and 10% positive bias for vendor C; all vendors exhibiting a ± 7% range of dispersion. Turning to the VFA measurements at 3 T, in Fig 5 we estimated these relative biases and dispersions as: 15% negative bias for vendor D in contrast to 7% positive bias for vendors C and E; all vendors exhibiting a ± 10% range of dispersion. Applying this bias and dispersion to the *T*_1_ values reported by de Blank et al. [8] results in *T*_1_ measurements that could be expected as per our current study (details in S1 File). We plotted the expected measurements alongside the reported ranges in Fig 7. The range of errors measured using IR is small, while the range of errors measured using VFA is significantly greater. If sites using vendor E wished to implement a threshold determination between LGG and GBM using *T*_1_ IR, it could be reasonable to do so by shifting the threshold based on the observed measurement bias. Similarly, if sites using vendors C and E wished to implement the threshold using *T*_1_ VFA, it may be reasonable to shift by the observed measurement bias. However, concerning *T*_1_ measurement by VFA on vendor D, the dispersion of *T*_1_ values is so great as to make it impossible to distinguish between the LGG and GBM tissue types with any confidence. What is more concerning, if the underestimate of *T*_1_ VFA exhibited by vendor D is not taken into account, then one could inaccurately diagnose a glioblastoma as a low-grade glioma, an incorrect determination with serious impacts to patient management.

Across all measurements, reported temperature of either the MRI room or of the bulk water in the phantom ranged from 17.1°C to 23.3°C. Previous research demonstrated that the *T*_1_ of NiCl_2_ solutions vary by ± 4% over this experimental range [20]. Therefore, we expect that the variation of *T*_1_ due to temperature is negligible compared to other sources of measurement error (see S1 Fig for additional details).

## Discussion

This study examined two *T*_1_ methods, the reference standard (IR) and a commonly used approach (VFA) and demonstrated that quantitative MRI measurement of *T*_1_ is potentially subject to significant bias and variation. There was no consistent pattern of discrepancy between vendors, and as a result, clinicians are unable to translate a diagnostic threshold *T*_1_ value determined on one MRI system to other MRI systems. The ability to compare measured values to known *T*_1_ values in a phantom is critical for disentangling various sources of bias and variation.

We included a range of MRI systems representative of clinical practice and analyzed the deviations in measured *T*_1_ from the reference *T*_1_ values in the ISMRM/NIST system phantom. Previous studies, which found less significant variation in measured *T*_1_ across sites, used six or fewer MRI systems and were highly controlled, in some cases programming the exact same sequence across two platforms from a single vendor rather than using a product sequence [11,12,32]. Similar to studies undertaken by Bane et al. [17] and vanHoudt et al. [19], our study included multiple vendor systems and multiple systems within a vendor including product or platform variation, and software variations. This study included two vendors at 1.5 T and three vendors at 3 T with more equal representation across vendors than these previous efforts. Studies, such as this one, establish lower bounds on the range of errors that one could expect for in vivo measurements.

The largest variations and bias in *T*_1_ measurement were for VFA measurement at 3 T. We suspect that a sizable component of the error in the VFA measurement could be due to imperfect B_1_ fields and associated nonregular slice profile [33], as it is known that VFA measurements are very susceptible to this source of error [34,35]. Flip angle is directly proportional to B_1_ field strength, and relative error in *T*_1_ is approximately twice that of the relative error in flip angle. This factor of two holds as a rule of thumb over a wide range of *T*_1_, as reported by [27] and confirmed by our numerical experiments. For example, if the RF pulse implementation leads to an effective 10% under-rotation for all angles, e.g., a 20 degree flip angle is actually 18 degree and so on for all other angles in the VFA sequence, then *T*_1_ measurements would be offset by approximately 20% in the same direction, e.g., a 2000 ms *T*_1_ would be measured as approximately 1600 ms. This same relative error would occur for any other nominal *T*_1_, and over-rotation results in over-estimation of measured *T*_1_ with the same sensitivity factor. We note that the NiCl_2_ array is not at isocenter in the A/P direction, which can result in less homogeneous B_1_ and B_0_. B_1_ variation could reasonably explain the range of *T*_1_ biases observed in Fig 5 and their apparent correlation with location of the sample sphere within the scanner adds support to this theory. However, additional measurements including a B_1_ field map would be needed for a more conclusive analysis.

Lack of B_1_ maps is a primary limitation of this study. At the time of data collection, B_1_ mapping was not commonly available on all systems and was therefore omitted. Since this time, other groups have clearly demonstrated that *T*_1_ mapping via VFA requires a B_1_ map [10,16,36], and some vendor-supplied correction methods are available [37], though even recent multi-site studies were unable to implement a product B_1_ map sequence on all systems [32]. Without B_1_ maps integrated into product *T*_1_ VFA, it will be challenging to implement *T*_1_ VFA for diagnostic purposes, as demonstrated in our analysis in Fig 7.

This work sets the foundation to validate and provide traceability for advanced quantitative MRI methods. We note, one limitation of reference phantom studies is that they cannot be used to assess sensitivity of the measurement to physiological effects. Prior to in vivo work, future studies could use these reference phantoms to assess the stability of measurements to variations in sequence parameter changes (e.g., voxel sizes, matrix sizes) and to assess vendor-specific quantitative MRI methods.

## Conclusion

Longitudinal relaxation time is one example of a variety of quantitative MRI parameters that are potentially measurable using clinical MRI systems. We suggest establishing rigorous quality control procedures for quantitative MRI to promote confidence and stability in associated measurement techniques and to enable translation of measurement thresholds for diagnostic, disease progression, and treatment monitoring from the research center to the entire clinical community and back. Standard phantoms that are curated and have traceable uncertainties are an important component of the rigorous quality control procedures required to validate and provide uncertainties for qMRI methods. We note that similar calls have been made previously by other researchers [38,39], and we strongly support these efforts.

## Supporting information

S1 Fig NMR-measured *T*_1_ variation with temperature. Here we show the *T*_1,NMR_ variation with temperature as a percent deviation from the *T*_1,NMR_ at 20 C. Please note, these measurements are for a different batch of NiCl_2_ solutions than the phantoms used in this study. However, the solutions were made to the same specifications, and we believe this to be representative of the solutions in this study.
(TIF)

S1 File Details for calculations in Fig 7. Here we detail the analyses and calculations that resulted in Fig 7.
(PDF)

## References

[1] Bydder GM, Steiner RE, Young IR, Hall AS, Thomas DJ, Marshall J, et al. Clinical NMR imaging of the brain: 140 cases. AJR American journal of roentgenology. 1982;139(2):215–36. doi: 10.2214/ajr.139.2.215 6979874

[2] Komiyama M, Yagura H, Baba M, Yasui T, Hakuba A, Nishimura S, et al. MR imaging: possibility of tissue characterization of brain tumors using T1 and T2 values. AJNR American journal of neuroradiology. 1987;8(1):65–70. 3028112 PMC8334042

[3] Just M, Thelen M. Tissue characterization with T1, T2, and proton density values: results in 160 patients with brain tumors. Radiology. 1988;169(3):779–85. doi: 10.1148/radiology.169.3.3187000 3187000

[4] Kjaer L, Thomsen C, Gjerris F, Mosdal B, Henriksen O. Tissue characterization of intracranial tumors by MR imaging. In vivo evaluation of T1- and T2-relaxation behavior at 1.5 T. Acta radiologica. 1991;32(6):498–504. 1742132

[5] Newman S, Haughton VM, Yetkin Z, Breger R, Czervionke LF, Williams AL, et al. T1, T2 and proton density measurements in the grading of cerebral gliomas. European Radiology. 1993;3:49–52.

[6] Araki T, Inouye T, Suzuki H, Machida T, Iio M. Magnetic resonance imaging of brain tumors: measurement of T1. Work in progress. Radiology. 1984;150(1):95–8. doi: 10.1148/radiology.150.1.6689793 6689793

[7] Badve C, Yu A, Dastmalchian S, Rogers M, Ma D, Jiang Y, et al. MR Fingerprinting of Adult Brain Tumors: Initial Experience. AJNR American journal of neuroradiology. 2017;38(3):492–9. doi: 10.3174/ajnr.A5035 28034994 PMC5352493

[8] de Blank P, Badve C, Gold DR, Stearns D, Sunshine J, Dastmalchian S, et al. Magnetic Resonance Fingerprinting to Characterize Childhood and Young Adult Brain Tumors. Pediatr Neurosurg. 2019;54(5):310–8. doi: 10.1159/000501696 31416081 PMC12908223

[9] Bojorquez JZ, Bricq S, Acquitter C, Brunotte F, Walker PM, Lalande A. What are normal relaxation times of tissues at 3 T? Magnetic resonance imaging. 2017;35:69–80. doi: 10.1016/j.mri.2016.08.021 27594531

[10] Lee Y, Callaghan MF, Acosta-Cabronero J, Lutti A, Nagy Z. Establishing intra- and inter-vendor reproducibility of T1 relaxation time measurements with 3T MRI. Magnetic resonance in medicine: official journal of the Society of Magnetic Resonance in Medicine/Society of Magnetic Resonance in Medicine. 2019;81(1):454–65. doi: 10.1002/mrm.27421 30159953

[11] Gracien RM, Maiworm M, Bruche N, Shrestha M, Noth U, Hattingen E, et al. How stable is quantitative MRI?—Assessment of intra- and inter-scanner-model reproducibility using identical acquisition sequences and data analysis programs. NeuroImage. 2020;207:116364. doi: 10.1016/j.neuroimage.2019.116364 31740340

[12] Weiskopf N, Suckling J, Williams G, Correia MM, Inkster B, Tait R, et al. Quantitative multi-parameter mapping of R1, PD(*), MT, and R2(*) at 3T: a multi-center validation. Front Neurosci. 2013;7:95. doi: 10.3389/fnins.2013.00095 23772204 PMC3677134

[13] Sullivan DC, Obuchowski NA, Kessler LG, Raunig DL, Gatsonis C, Huang EP, et al. Metrology Standards for Quantitative Imaging Biomarkers. Radiology. 2015:142202. 10.1148/radiol.2015142202 PMC4666097 26267831

[14] Captur G, Gatehouse P, Keenan KE, Heslinga FG, Bruehl R, Prothmann M, et al. A medical device-grade T1 and ECV phantom for global T1 mapping quality assurance-the T1 Mapping and ECV Standardization in cardiovascular magnetic resonance (T1MES) program. J Cardiovasc Magn Reson. 2016;18(1):58. doi: 10.1186/s12968-016-0280-z 27660042 PMC5034411

[15] Zhang Q, Werys K, Popescu IA, Biasiolli L, Ntusi NAB, Desai M, et al. Quality assurance of quantitative cardiac T1-mapping in multicenter clinical trials—A T1 phantom program from the hypertrophic cardiomyopathy registry (HCMR) study. Int J Cardiol. 2021;330:251–8. doi: 10.1016/j.ijcard.2021.01.026 33535074 PMC7994017

[16] Stikov N, Boudreau M, Levesque IR, Tardif CL, Barral JK, Pike GB. On the accuracy of T1 mapping: searching for common ground. Magnetic resonance in medicine: official journal of the Society of Magnetic Resonance in Medicine/Society of Magnetic Resonance in Medicine. 2015;73(2):514–22. doi: 10.1002/mrm.25135 24578189

[17] Bane O, Hectors SJ, Wagner M, Arlinghaus LL, Aryal MP, Cao Y, et al. Accuracy, repeatability, and interplatform reproducibility of T1 quantification methods used for DCE-MRI: Results from a multicenter phantom study. Magnetic resonance in medicine: official journal of the Society of Magnetic Resonance in Medicine/Society of Magnetic Resonance in Medicine. 2018;79(5):2564–75. 10.1002/mrm.26903 PMC5821553 28913930

[18] Keenan KE, Gimbutas Z, Dienstfrey A, Stupic KF. Assessing effects of scanner upgrades for clinical studies. Journal of magnetic resonance imaging: JMRI. 2019;50(6):1948–54. doi: 10.1002/jmri.26785 31111981

[19] van Houdt PJ, Kallehauge JF, Tenderup K, Nout R, Zaletelj M, Tadic T, et al. Phantom-based quality assurance for multicenter quantitative MRI in locally advanced cervical cancer. Radiotherapy and Oncology. 2020. doi: 10.1016/j.radonc.2020.09.013 32931890

[20] Stupic KF, Ainslie M, Boss MA, Charles C, Dienstfrey AM, Evelhoch JL, et al. A standard system phantom for magnetic resonance imaging. Magnetic resonance in medicine: official journal of the Society of Magnetic Resonance in Medicine/Society of Magnetic Resonance in Medicine. 2021. doi: 10.1002/mrm.28779 33847012 PMC8252537

[21] Boss MA, Dienstfrey AM, Gimbutas Z, Keenan KE, Kos AB, Splett JD, et al. Magnetic Resonance Imaging Biomarker Calibration Service: Proton Spin Relaxation Times. NIST Special Publication 250–97. National Institute of Standards and Technology; 2018. Report No.: 97 Contract No.: SP-250-97.

[22] Chenevert TL, Malyarenko DI, Newitt D, Li X, Jayatilake M, Tudorica A, et al. Errors in Quantitative Image Analysis due to Platform-Dependent Image Scaling. Translational oncology. 2014;7(1):65–71. doi: 10.1593/tlo.13811 24772209 PMC3998685

[23] Cabana JF, Gu Y, Boudreau M, Levesque IR, Atchia Y, Sled JG, et al. Quantitative Magnetization Transfer Imaging Made Easy with qMTLab: Software for Data Simulation, Analysis, and Visualization. Concept Magn Reson A. 2015;44a(5):263–77.

[24] Karakuzu A, Boudreau M, Duval T, Leppert I, Boshkovski T, Pike GB, et al. qMRLab [http://qmrlab.org.

[25] Quantitative Magnetic Resonance Imaging. 1st ed: Academic Press; 2020. 1092 p.

[26] Ernst FJ, Warnock RL, Wali KC. Linear and Nonlinear Mass-Difference Effects in a Model of Baryon Multiplets. Phys Rev. 1966;141(4):1354-+.

[27] Helms G, Dathe H, Weiskopf N, Dechent P. Identification of signal bias in the variable flip angle method by linear display of the algebraic Ernst equation. Magnetic resonance in medicine: official journal of the Society of Magnetic Resonance in Medicine/Society of Magnetic Resonance in Medicine. 2011;66(3):669–77. doi: 10.1002/mrm.22849 21432900 PMC3193384

[28] Kessler LG, Barnhart HX, Buckler AJ, Choudhury KR, Kondratovich MV, Toledano A, et al. The emerging science of quantitative imaging biomarkers terminology and definitions for scientific studies and regulatory submissions. Stat Methods Med Res. 2015;24(1):9–26. doi: 10.1177/0962280214537333 24919826

[29] Page MC, Braver SL, MacKinnon DP. Levine’s guide to SPSS for analysis of variance. 2nd ed: Lawrence Erlbaum Associates Publishers; 2003.

[30] Dunn OJ, Clark VA. Applied Statistics: Analysis of Variance and Regression. New York: Wiley; 1974.

[31] Kirk RE. Experimental Design: Procedures for the Behavioral Sciences. 3rd ed. Monterey, CA: Brooks/Cole Publishing; 1995.

[32] Leutritz T, Seif M, Helms G, Samson RS, Curt A, Freund P, et al. Multiparameter mapping of relaxation (R1, R2*), proton density and magnetization transfer saturation at 3 T: A multicenter dual-vendor reproducibility and repeatability study. Hum Brain Mapp. 2020. 10.1002/hbm.25122 PMC7502832 32639104

[33] Zheng J, Venkatesan R, Haacke EM, Cavagna FM, Finn PJ, Li D. Accuracy of T1 measurements at high temporal resolution: feasibility of dynamic measurement of blood T1 after contrast administration. Journal of magnetic resonance imaging: JMRI. 1999;10(4):576–81. 10508325 10.1002/(sici)1522-2586(199910)10:4<576::aid-jmri11>3.0.co;2-p

[34] Tsai WC, Kao KJ, Chang KM, Hung CF, Yang Q, Lin CE, et al. B1 Field Correction of T1 Estimation Should Be Considered for Breast Dynamic Contrast-enhanced MR Imaging Even at 1.5 T. Radiology. 2017;282(1):55–62. doi: 10.1148/radiol.2016160062 27479805

[35] Wang J, Qiu M, Constable RT. In vivo method for correcting transmit/receive nonuniformities with phased array coils. Magnetic resonance in medicine: official journal of the Society of Magnetic Resonance in Medicine/Society of Magnetic Resonance in Medicine. 2005;53(3):666–74. doi: 10.1002/mrm.20377 15723397

[36] Lee Y, Callaghan MF, Nagy Z. Analysis of the Precision of Variable Flip Angle T1 Mapping with Emphasis on the Noise Propagated from RF Transmit Field Maps. Front Neurosci. 2017;11:106. doi: 10.3389/fnins.2017.00106 28337119 PMC5343565

[37] Bliesener Y, Zhong X, Guo Y, Boss M, Bosca R, Laue H, et al. Radiofrequency transmit calibration: A multi-center evaluation of vendor-provided radiofrequency transmit mapping methods. Medical physics. 2019;46(6):2629–37. doi: 10.1002/mp.13518 30924940 PMC6598716

[38] Hanson CA, Kamath A, Gottbrecht M, Ibrahim S, Salerno M. T2 Relaxation Times at Cardiac MRI in Healthy Adults: A Systematic Review and Meta-Analysis. Radiology. 2020;297(2):344–51. doi: 10.1148/radiol.2020200989 32840469 PMC7605362

[39] Partridge SC, Zhang Z, Newitt DC, Gibbs JE, Chenevert TL, Rosen MA, et al. Diffusion-weighted MRI Findings Predict Pathologic Response in Neoadjuvant Treatment of Breast Cancer: The ACRIN 6698 Multicenter Trial. Radiology. 2018:180273. doi: 10.1148/radiol.2018180273 30179110 PMC6283325

