# Supplementary material for: Multi-site, multi-platform comparison of MRI T1 measurement using the system phantom
Source: PLoS One. 2021 Jun 30;16(6):e0252966. doi: 10.1371/journal.pone.0252966 (PMC8244851; doi:10.1371/journal.pone.0252966)
Supplement: S1 File — Here we detail the analyses and calculations that resulted in Fig 7. (PDF) [file pone.0252966.s002.pdf]

S1 File. Details for calculations in Fig 7.

First, we assumed that a reasonable diagnostic criteria based on the data in (8) was the center half of the reported interval for low grade gliomas and glioblastomas (i.e., $T_{1,\mathrm{LGG}}$ diagnostic range is 1262 ms to 1449 ms, and $T_{1,\mathrm{GBM}}$ diagnostic range is 1828 ms to 1898 ms).

We assumed that the biases and dispersions of MRI T_1 measurement techniques were constant over the range of T_1 required by this scenario, ~1200 ms to ~1900 ms. These constants were determined using measurement data from the two $NiCl_2$ array samples closest to this diagnostic range. In this case, these are the two longest values, $T_{1,\mathrm{NMR}}$ = 1489 ms and $T_{1,\mathrm{NMR}}$ = 2033 ms.

We used Markov chain Monte Carlo algorithm (Gibbs sampling) to estimate the distribution of measured T_1 times that would result at 3 T for each vendor and measurement technique (IR and VFA) when presented with a distribution of T_1 times lying within the low-grade glioma and glioblastoma diagnostic intervals. For each of these low-grade glioma and glioblastoma scenarios, we assumed that the true T_1 is a uniform random variable with endpoints as per the diagnostic criteria. For any true T_1 drawn from this interval, we assume that the T_1 as measured using qMRI will be normally distributed with a mean and variance defined by applying the bias and dispersion factors determined by our study. The probability density for the MRI T_1 value cannot be expressed in closed form; however, its conditional structure allows for efficient sampling. For all cases, we drew 10000 samples and used these to compute the means and 95 % confidence intervals for the distribution of T_1 measurements.
